# Experimentally controlled study indicates that the naturally occurring recombinant vaccine-like lumpy skin disease strain Udmurtiya/2019, detected during freezing winter in northern latitudes, is transmitted *via* indirect contact

**DOI:** 10.3389/fvets.2022.1001426

**Published:** 2022-10-20

**Authors:** Alexander Nesterov, Ali Mazloum, Olga Byadovskaya, Irina Shumilova, Antoinette Van Schalkwyk, Alena Krotova, Vladimir Kirpichenko, Irina Donnik, Ilya Chvala, Alexander Sprygin

**Affiliations:** ^1^Federal Center for Animal Health, Vladimir, Russia; ^2^Agricultural Research Council—Onderstepoort Veterinary Institute, Pretoria, South Africa; ^3^Department of Biotechnology, University of the Western Cape, Bellville, South Africa; ^4^Kazakh Scientific Research, Veterinary Institute, Almaty, Kazakhstan; ^5^Russian Academy of Sciences, Moscow, Russia

**Keywords:** lumpy skin disease virus, infection, transmission, epidemiology, recombination

## Abstract

Lumpy skin disease (LSD) caused by LSD virus (LSDV), is a member of the poxvirus genus *Capripoxvirus*. It is classified as a notifiable disease by the World Organization for Animal Health (WOAH) based on its potential for rapid spread and global economic impact. Due to these characteristics, the mode of LSDV transmission has prompted intensive research efforts. Previous experimental studies using the virulent vaccine-derived recombinant LSDV strain Saratov/2017, demonstrated that this strain has the capacity for transmission in a vector-proof environment. This study demonstrated that a second novel recombinant vaccine-derived LSDV strain Udmurtiya/2019, can infect bulls in contact with diseased animals, in the absence of insect vectors. Bulls were housed in an insect proof animal biosafety level 3 facility, where half the animals were inoculated intravenously with the recombinant LSDV (Udmurtiya/2019), whilst the remaining five animals were mock-inoculated but kept in contact with the inoculated group. Both the infected / inoculated group (IN) and uninfected / incontact group (IC), were monitored for 41 days with continuous registration of body temperature, observations for clinical signs and collection of blood samples and nasal swabs for testing of LSDV presence using real-time PCR. Results indicated that cohabitation of animals from both groups was sufficient to transmit the virus from the IN to the IC-group, with the onset of clinical signs including pyrexia (~41°C) and classical LSD nodular skin lesions starting at 10 dpi for the IN group and 16 dpi for the IC-group. Additionally, the presence of LSDV genomes as well as anti-LSDV antibodies were detected in swabs, blood and serum samples from animals belonging to both groups. These results provides additional evidence of LSDV transmission in a controlled environment without direct contact between diseased and healthy animals, yet in the absence of vectors. Based on these observations, the question concerning a hypothetical relation between mutations in the virus genome and its mode of transmission gains more importance and requires additional investigations with direct comparisons between classical and novel recombinant LSDV strains.

## Introduction

Lumpy skin disease (LSD) is a viral disease of cattle and water buffaloes, belonging to the poxvirus genus *Capripoxvirus*. It is classified as a notifiable disease by the World Organization for Animal Health (WOAH) based on its potential for rapid spread and impact on the global economy (1).

The causative agent of LSD, lumpy skin disease virus (LSDV), is oval shaped with large lateral bodies and an average size of 320 × 260 nm. It contains a double-stranded DNA (dsDNA) genome ~151 kb in size, encoding 156 open reading frames (ORFs) (2). The LSDV genes share a significant percentage sequence identity (at least 96%) with genes from other poxviruses of the genus *Capripoxvirus*, namely, sheep pox virus (SPPV) and goat pox virus (GTPV) (3).

In the previous century, LSDV circulation was predominantly confined to Africa and later the Middle East. However, in the last decades the geographical range of LSDV has expanded dramatically into countries in the northern hemisphere, from where the disease was previously absent (4). The first reports of LSD in the Balkan countries, Greece, and Russia were between 2015 and 2016 (4). The Russian Federation (RF) reported annual LSD outbreaks from 2015 to 2020, with a dramatic shift in the LSDV strains responsible for these outbreaks (5). Since 2019, China, India and other counties of South Asia have been affected by LSDV (6, 7).

Vaccination is an effective strategy to prevent and control LSD outbreaks and currently two types of vaccines are globally administered against LSD: homologous or heterologous vaccines (8). Heterologous LSD vaccines are produced from attenuated strains of SPPVs and GTPVs, employing the principle of cross protection. This is based on the serological and genetic relatedness of capripox viruses, despite their individual host-restrictions, i.e., bovines, ovines and caprines associated with LSDV, SPPV and GTPV respectively (9). Homologous vaccines are produced from attenuated LSDV strains, with the predominantly used vaccines based on either the Neethling-LW1959 of Kenyan sheep and goat pox (KSGP) strains (8). In contrast to the heterologous vaccines that do not replicate in cattle, the homologous vaccines are prone to genetic drift and recombination similar to virulent LSDVs capable of replication in bovines (10, 11).

Poxviruses are commonly transmitted through direct contact rather than mechanical transmission using arthropod vectors, for example virus contaminated insects either biting or feeding on susceptible hosts (12). In contrast to the aerosol transmission pathways employed by both SPPV and GTPV, initial studies suggested that contact transmission was an ineffective route for LSDV (13–15). Based on the seasonality of LSD outbreaks it was considered a vector-borne disease that spread mechanically *via* insect bites (15). However, non-vector mediated in-contact transmission was observed for the first naturally occurring recombinant vaccine-like LSDV strain, Saratov/2017 (16).

The genome of Saratov/2017 is composed of the Neethling-LW1959 live attenuated commercial vaccine strain as the major parent and a Kenyan vaccine strain (KSGPO240—like) as the minor parent (17). The occurrence of recombinant strains was first documented in 2017 in Saratov, Russia, following a national vaccination campaign with the Kenyan vaccine KEVIVAP in Kazakhstan. The LSDV, Saratov/2017, was the first of many novel recombinants between vaccine-derived parental strains to be isolated from active LSD outbreaks in the field in Russia, Kazakhstan and China (18). Subsequent experiments using Saratov/2017 provided evidence that this recombinant vaccine-like strain is capable of infecting bulls *via* indirect contact and virus-inoculated feed, i.e., *via* the alimentary route, thus corroborating its contagious nature and resembling the transmission pathway described in the majority of poxviruses (16, 19, 20).

Since the isolation of Saratov/2017, all LSD outbreaks in the RF were due to novel recombinant vaccine-like strains (5). Outbreaks have been reported to occur below freezing temperatures, thus inhibiting the optimal flight activity of the insects previously identified in the temperate climate of the RF (5). An outbreak of LSD detected during the freezing winter in the Republic of Udmurtiya in the RF, provided additional support for potential alternative modes of LSDV transmission in addition to vector-borne transmission (21). A study into the genomic composition of the novel recombinant vaccine-like strain Udmurtiya/2019 described that although the parental strains are the same as Saratov/2017, i.e., the Neethling-LW1959 and KSGPO-240-like vaccine strains, its contributions of parental material were different (21). The Udmurtiya/2019 genomic backbone is composed of KSGPO-240 vaccine strain as the major component and Neethling-LW1959 as the minor parent (18). The freezing conditions prevailing during the detection of the novel recombinant Udmurtiya/2019, together with the unique mosaic genomic structure of virus, necessitates additional experimental evaluation in order to expand our knowledge of the biological properties associated with the novel recombinant vaccine-like strain. This is especially of importance, since novel recombinant strains are the dominant LSDV lineage circulating in Russia and Asia (18).

In this paper we provide new evidence indicating that the novel recombinant vaccine-derived LSDV strain, Udmurtiya/2019, with reversed parental combinations compared to Saratov/2017, can infect in-contact bulls in the absence of insect vectors, thus it is capable of transmission through alternative in-contact methods.

## Materials and methods

### Virus

A recombinant vaccine-like strain of lumpy skin disease virus was isolated during the freezing winter in Russia in 2019, from bovines presenting with severe clinical signs of LSD (21). The isolate was subjected to two serial passages in goat testis cells, prior to the characterization of the LSDV through polymerase chain reaction (PCR) amplification of different loci specific to either vaccine or field strain genomes (22). Virus titration was performed in 96-microwell plates, using 10-fold dilution. The plates were incubated at 37°C with 5% CO_2_ for 72 h and inspected daily for the presence of a cytopathic effect (CPE). Cell or negative control wells had to demonstrate the absence of CPE, whilst characteristic CPE in the form of lumps on the cell layer had to be present in wells for the virus or positive control. Virus titer was calculated according to the Spearman-Karber method and as reported previously (16). The results are expressed in logarithm as 50% tissue culture infective dose (log TCID50).

### Ethics statement

The animal experiment, as well as the euthanasia procedure, were approved by the Ethics Committee of the Federal Center for Animal Health, Russia (Permit Number: No. 2/1-21082018) and conducted in strict accordance with Directive 2010/63/EU on the protection of animals used for scientific purposes. The euthanasia protocol (permit number No 2/03-15022022, provided as approval by the ethical committee of FGBI ARRIAH) consisted of captive-bold penetration to desensitize the animals, followed by the injection of the muscle relaxant, Adilinum super (Federal Center for Toxicological, Radiation and Biological Safety, Kazan, Russia) at 41 days post infection (dpi). The latter is administered at the recommended dose of 5 mg/kg according to the drug use instruction approved by the Russian Federal Service for Veterinary and Phytosanitary Surveillance in 2008. At the recommended dose, the Adilinum mechanism of action provides painless and rapid euthanasia: cerebral death commences first followed by circulatory collapse.

### Experimental design

The experiment included ten Russian Black Pied breed bulls aged 6–8 months, weighing between 300 and 500 kg. Animals were randomly numbered from 1 to 10 and housed in an insect proof animal Biosafety Level 3 facility with a 12-hourly light-dark cycle, relative humidity of 30% to 70% and temperature range between 23 and 26°C. All animals were monitored twice a day by the veterinary staff. Water and feed were provided *ad libitum*. Since the experiment was performed in an insect-proof facility, the possible presence of any dipteran insects was detected by indoor blood-feeding insect UV light traps and sticky traps mounted at regular intervals on the walls of the facility. The animals were also examined for the presence of ticks. Animals were kept in the facility for 2 weeks prior to the start of the experiment, in order to adapt to the conditions, whilst blood samples and nasal swabs were obtained for PCR and neutralization tests to exclude previous or present LSDV infections.

The five animals with odd numbers (1, 3, 5, 7 and 9) each received 2 ml of 5 log TCID50/ml of the recombinant virus, LSDV Udmurtiya/2019, intravenously. These five animals were subsequently called the infected or inoculated group (IN). The remaining five animals with even numbers were not inoculated but kept in contact with IN animals. This group was called the in-contact (IC) animals (Table 1). The animals were housed in the same ventilated insect-proof facility where they could see each other, but any physical contact between them as well as sharing of water troughs, food or bedding were prohibited. Their mobility was restricted using tethering. Virus inoculations were performed on 0 dpi and the experiment lasted for 41 day, followed by humane euthanasia.

**Table 1:** Schematic of animal allocation.

**No. animals**									
**IN-1**	**IC-2**	**IN-3**	**IC-4**	**IN-5**	**IC-6**	**IN-7**	**IC-8**	**IN-9**	**IC-10**
+	–	+	–	+	–	+	–	+	–

“+”, virus inoculated animals; “–”, in-contact animals.

The animals were monitored daily for the presence of fever and clinical signs of LSD until the end of the experiment at 41 days. In order to evaluate the disease development over time, especially virus shedding and viremia, nasal discharges, skin scabs and blood were collected every second day until day 41. The samples were submitted for real-time PCR to detect the presence of LSDV nucleic acids.

### Virus isolation in cell culture

The skin lesions and nasal swabs were collected from infected animals using sterile saline and a 10% suspension (m/v) were prepared. The samples were subjected to three freeze-thaw cycles at−80°C and room temperature (RT), in order to disrupt the cell membrane and release virus. The suspension was clarified by centrifugation at 2,000 g for 15 mins (min) and the supernatant was removed to a new tube. Antibiotics (penicillin and streptomycin at final concentrations of 2,000 IU/mL and 2 mg/mL, respectively) were added, incubated at RT for 90 min and 0.3 ml was used to inoculate ovine testis or goat gonad cells (70-80 % confluent cell layer) cultured in T-25 flasks. Growth medium from flasks containing the cell cultures was removed and cells were washed twice with Hanks' medium. A 0.3 mL volume of inoculum was added and the flasks were incubated at 37°C for 90 min to ensure virus adhesion, followed by addition of 10 mL maintenance medium supplemented with 0.2 ml fetal bovine serum (FBS) and incubated at 37°C. The inoculated cell cultures were observed daily for cytopathic effect (CPE). The cells were harvested when 80% CPE was observed and lysed to release the virus, using three repeated freeze- thaw cycles. The presence of LSDV was confirmed using real-time PCR.

### Virus neutralization

Virus neutralization in flat-bottomed microplates (96 wells) was conducted using the protocol previously described (23), with a few modifications. The test was performed on ovine testis cells with two replicates. The volume of virus inoculum was 100 μl into each well and the neutralization index was considered negative if ≤1:8.

### DNA extraction

The samples were aseptically handled and processed as 10% homogenates in PBS. A 200 μL aliquot was used for total nucleic acid extraction using the QIAamp DNA Mini Kit (Qiagen, Germany), following the manufacturer's recommendations.

### Real-time PCR (quantitative QPCR)

Sample extracts were analyzed for the presence of LSDV DNA using real-time PCR based on ORF044 as previously described (22). The fluorogenic probe was labeled at the 5′ end with the FAM reporter dye and with BHQ as a quencher at the 3′ end. Selected primers (df4ln: CAAAAACAATCGTAACTAATCCA and zdr4ln: TGGAGTTTTTA TGTCATCGTC) and probes (zdpro4ln1: Fam-TCGTCGTCGTTTAAAACTGA-BHQ1) were synthesized by Syntol (Moscow, Russia). PCR was performed using a Rotor-Gene Q (Qiagen, Germany) instrument and the following thermal-cycling profile: 95°C for 10 min, followed by 45 cycles at 95°C for 15 seconds (s) and 60°C for 60 s. The final reaction volume was 25 μL containing 10 pmol of each primer, as well as 5 pmol of the probe, 5μL of 25 mM MgCl2, 5 μL 5 × PCR Buffer (Promega, USA), 1 μL of 10 pmol dNTPs (Invitrogen, USA), and deionized water to make up the final volume. Samples were tested and results interpreted according to the protocol, as previously described (22).

## Results

### Body temperature

**Figure 1:**
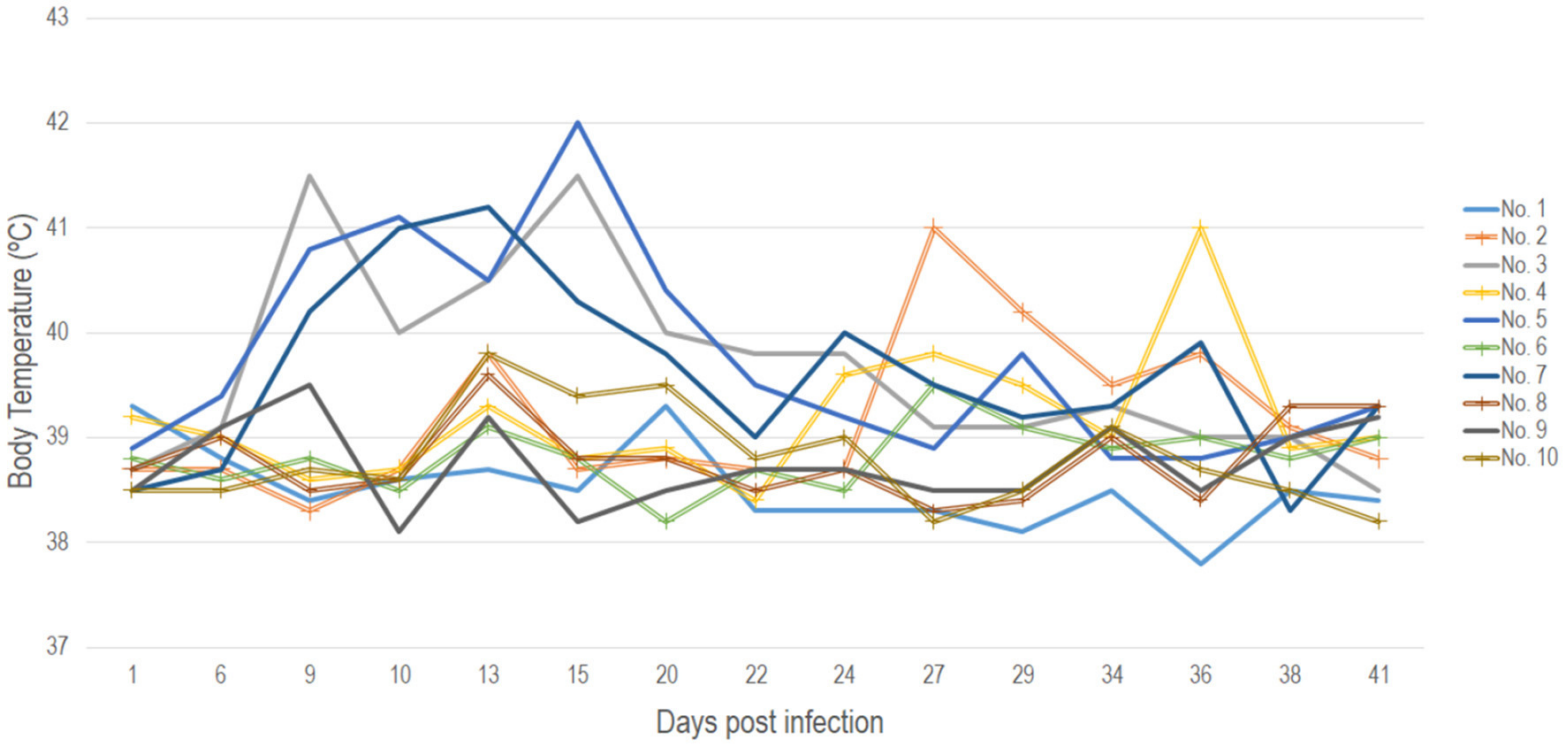
Recorded daily body temperatures for all animals in both the IN (points not marked) and IC (points marked with a cross) groups throughout the 41 days duration of the experiment.

The baseline average temperature for the experimental animals prior to the start of the trial was 39.5°C. On the 8th dpi three of the five animals belonging to the IN group displayed a fever reaching 42.0°C (range 39.6 to 42.0°C) (Figure 1). Animals in the IC group maintained normal body temperature of around 38.6°C until day 13 pi when animals No. 2, No. 8 and No. 10 recorded a temperature of 39.6°C. The temperature of these animals normalized again, until day 23 pi when bull No. 4 had a fever of 39.6°C and 3 days later bull No. 2 had a fever of 41.0 °C which lasted for 10 days (Figure 1). Full results of the recorded body temperature for all the animals in both IC and IN groups are presented in Figure 1.

### Clinical manifestation

**Figure 2:**
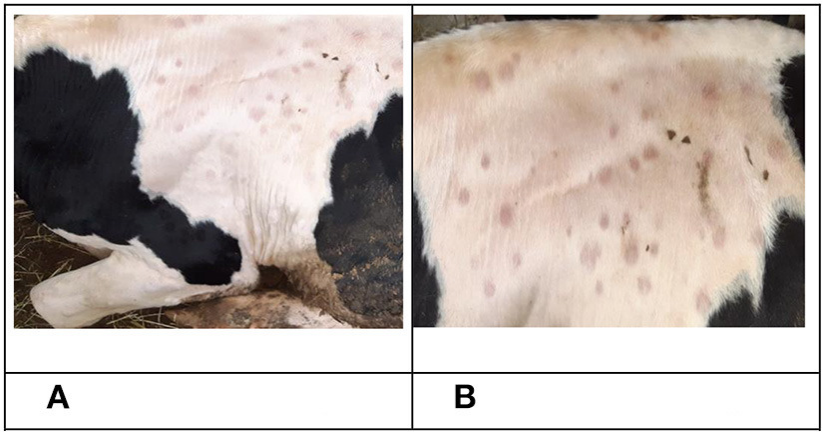
Clinical manifestation of LSDV in bull No. 5 belonging to the IN group. **(A)** Nodular skin lesions, 10 dpi. **(B)** Nodular skin lesions, 16 dpi.

The first clinical manifestations of LSD in the form of small skin nodules on the neck and shoulder area, as well as roseola on the scrotum were observed on days eight and nine pi. in animals No. 3, No. 5 and No. 7 belonging to the IN group. This was simultaneous to the previously mentioned increase in temperature (Figure 1). On the 10th day, bull No. 5 was in a state of apathy, displaying a refusal to move and multiple nodules throughout its body (Figures 2A,B). Animals No. 3, No. 5 and No. 7 displayed an increase in superficial lymph nodes, weakness and heavy breathing, whilst on the 11th dpi these skin lesions reached between 0.3 × 0.3 cm to 4.0 × 4.5 cm in size and covered the entire body.

Additionally, on the 11th dpi the first clinical signs of LSD were detected in the IC group with several small nodules, about 0.2–0.3 cm in diameter, forming on the left shoulder blade and left side of the neck of bull No.8.

**Figure 3:**
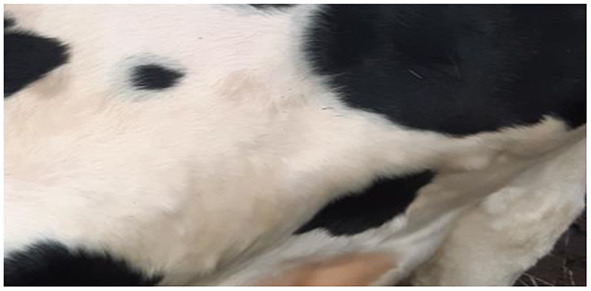
Nodular skin lesions observed on bull No. 8, belonging to the IC group, 16 dpi.

**Figure 4:**
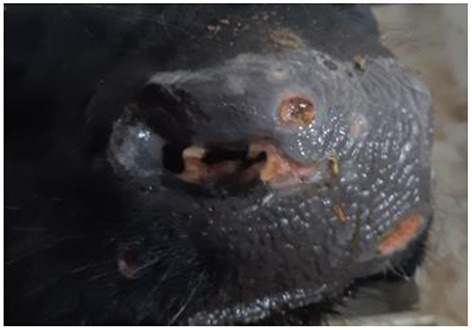
Erosive skin lesions of the nasolabial speculum of bull No. 3, belonging to the IN group, 20 dpi.

Five days later (16 dpi), the number and size of nodular lesions increased, reaching a diameter of 0.5–0.7 cm (Figure 3). Additional small nodular lesions were detected on the clavicle area of the same animal (bull No.8), on day 16 dpi. During this time, inflamed nodular lesions were observed in infected animals (No. 3, No. 5 and No. 7) in addition to erosive skin lesions of the nasolabial speculum (Figure 4).

**Figure 5:**
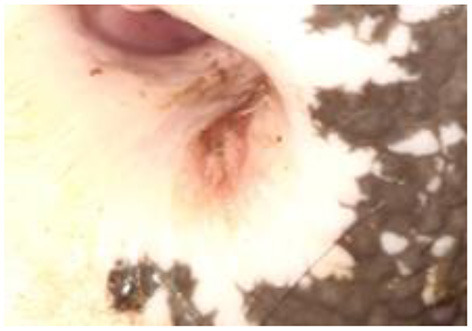
Erosive lesions of the nasal mucosa of bull No 6, belonging to the IC group, on 28 dpi.

**Figure 6:**
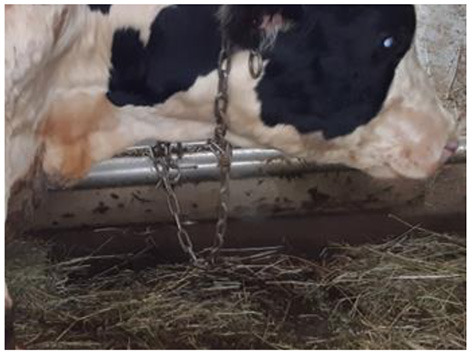
Edema in the submandibular region of bull No. 4, belonging to the IC group, 30 dpi.

**Figure 7:**
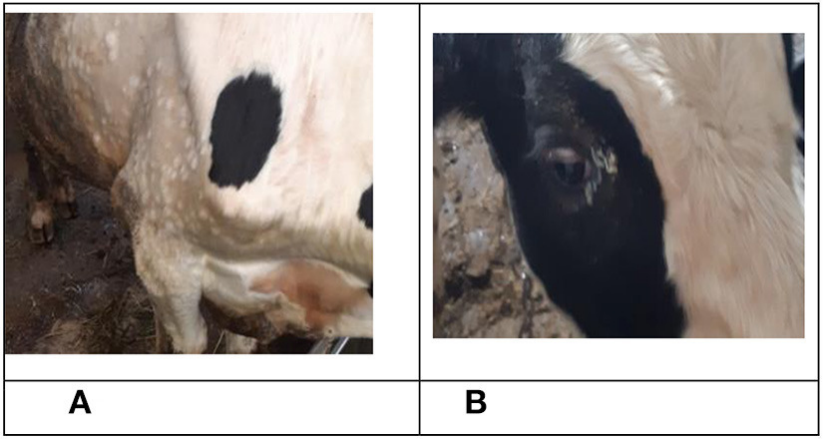
Clinical manifestation of LSDV in bull No. 2, belonging to the IC group. **(A)** Nodular skin lesions, 32 dpi. **(B)** Purulent conjunctivitis 34 dpi.

Clinical manifestations of LSD were observed in four of the five uninfected (IC) animals on day 26 of the experiment. Bulls No.2, No.4, and No.6 displayed severe symptoms including nodular lesions on their backs, heads, fore and hind limbs, scrotum, erosive lesions on the nasolabial mirror, enlarged lymph nodes and subcutaneous tissue edema in the submandibular region (Figures 5, 6, 7A).

In contrast, bull No. 8, displayed a few small-nodular skin lesions on the left shoulder blade and left side of its neck for 10 days, followed by no additional external manifestations of LSD.

The infected animals (No.3, No.5, and No.7), continuously displayed clinical signs of LSD as previously described, until the end of the experiment on day 42. Additionally, bull No.7 developed painful edema on the right hind leg, severe weakness, decreased appetite and exhaustion.

Uninfected animals No.2, No.4, and No.6 developed classical LSD symptoms during the progression of the experiment. On day 33, bull No.2 developed conjunctivitis (Figure 7B), whilst on day 36 conjunctivitis secretions were sampled from this animal for real time PCR testing to confirm the presence of LSDV. The qPCR results indicated the presence of LSDV genome with a Ct value of 30.15.

Severe disease symptoms were observed in bull No.4, which included extensive edema on the left half of the animal's head.

The animals No.1 and No.9, which were intravenously infected with LSDV Udmurtiya/2019 at the start of the experiment, as well as one of the control animals, No.10, remained healthy and without the visible signs of clinical LSD for the 41 days duration of the experiment.

### RT-PCR

LSDV DNA was detected in all animals that received the inoculum (IN group) with varying viremia. The viremia lasted for 16–32 days from day 6 to day 41 except for bull No1 that was transiently positive at day 20 pi only (Tables 2, 3).

**Table 2:** Real time PCR results detecting the presence of LSDV in blood (Ct values).

**No. bull**	**Day post infection (dpi)**													
	**1**	**6**	**9**	**13**	**15**	**20**	**22**	**24**	**27**	**29**	**34**	**36**	**38**	**41**
1	Nd	Nd	Nd	Nd	Nd	**34,31**	Nd	Nd	Nd	Nd	Nd	Nd	Nd	Nd
2	Nd	Nd	Nd	Nd	Nd	Nd	Nd	**33,05**	**22,59**	**27,84**	**17,23**	**26,52**	Nd	**30,45**
3	Nd	Nd	Nd	**23,78**	**19,15**	**24,10**	**25,32**	**28,52**	**29,64**	**33,56**	**34,51**	Nd	**33,39**	**30,02**
4	Nd	Nd	Nd	Nd	Nd	Nd	Nd	**32,91**	**27,85**	**26,86**	**25,20**	**26,11**	**26,84**	**25,56**
5	Nd	**32,32**	**25,18**	**20,26**	**24,78**	**23,92**	**24,87**	**25,72**	**24,77**	**29,62**	**32,75**	Nd	Nd	Nd
6	Nd	Nd	Nd	Nd	Nd	Nd	**34,01**	**32,15**	**27,72**	**34,29**	**27,53**	**29,62**	**30,49**	Nd
7	Nd	Nd	**24,78**	**22,86**	**20,99**	**22,52**	**23,65**	**24,46**	**25,43**	**27,52**	**27,88**	**30,61**	**34,25**	**30,05**
8	Nd	Nd	Nd	Nd	Nd	Nd	Nd	Nd	Nd	Nd	Nd	Nd	Nd	Nd
9	Nd	Nd	Nd	**29,07**	Nd	**30,58**	**31,23**	**31,01**	**33,80**	**34,27**	Nd	Nd	Nd	Nd
10	Nd	Nd	Nd	Nd	Nd	Nd	Nd	Nd	Nd	Nd	Nd	Nd	Nd	Nd

Ct values <35 are considered positive; Ct values between 35 and 40 are inconclusive; Not detected (Nd) implies a negative result. Bold means positive.

**Table 3:** Real time PCR results detecting the presence of LSDV in nasal swabs (Ct values).

**No. bull**	**Day post infection (dpi)**													
	**1**	**6**	**9**	**13**	**15**	**20**	**22**	**24**	**27**	**29**	**34**	**36**	**38**	**41**
1	Nd	Nd	Nd	Nd	Nd	**26,56**	Nd	Nd	Nd	Nd	Nd	Nd	Nd	Nd
2	Nd	Nd	Nd	Nd	**35,08**	**33,64**	**33,65**	**30,78**	**24,35**	**17,54**	**25,70**	**22,16**	**20,71**	**25,02**
3	Nd	Nd	Nd	**15,49**	**14,27**	**16,48**	**14,50**	**21,66**	**22,21**	**25,34**	**28,52**	**24,89**	**28,93**	**30,65**
4	Nd	Nd	Nd	Nd	**27,33**	**19,49**	**21,04**	**18,61**	**24,12**	**25,65**	**30,48**	**27,70**	**30,32**	**29,83**
5	Nd	Nd	**27,33**	**19,49**	**21,04**	**18,61**	**24,12**	**25,65**	**30,48**	**27,70**	**30,32**	**29,83**	Nd	**32,08**
6	Nd	Nd	Nd	Nd	Nd	Nd	**33,72**	**34,72**	**28,98**	**26,03**	**29,35**	**23,81**	**24,18**	**24,52**
7	Nd	Nd	**29,00**	**17,79**	**22,26**	**20,20**	**25,07**	**19,27**	**29,24**	**21,25**	**22,92**	**26,65**	**29,41**	Nd
8	Nd	Nd	Nd	Nd	Nd	Nd	**31,98**	**33,10**	**30,46**	**32,42**	**32,13**	**30,62**	Nd	**32,79**
9	Nd	Nd	Nd	**23,78**	Nd	Nd	**31,86**	Nd	Nd	Nd	**33,42**	**33,98**	Nd	Nd
10	Nd	Nd	Nd	Nd	Nd	Nd	Nd	Nd	Nd	Nd	Nd	Nd	Nd	**32,97**

Ct values <35 are considered positive; Ct values between 35 and 40 are inconclusive; Not detected (Nd) implies a negative result. Bold means positive.

Results from the nasal swab samples indicated that all the animals belonging to the IN group either exhibited virus shedding or were exposed to virus for 19-32 days, starting from day 9 until 41 dpi (Table 3). Bull No1 was again transiently positive at day 20 pi, which could indicate new exposure to the virus (Table 3).

Similarly, all the IC-group produced positive PCR results when using DNA extracted from nasal swab samples, albeit for bull No 10 this only occurred on day 41 (Table 3). These PCR positive results were obtained from day 15 pi and lasted a maximum of 26 days (Table 3). In contrast, PCR using DNA extracted from blood samples were positive from as early as day 22 pi and lasted for 17 days (Table 2). The PCR with template extracted from the discharge of the infected eye of bull No2, also produced a positive result. In-contact bulls No8 and No10 had no detectable viremia, despite showing exposure to the virus by positive PCRs using DNA from nasal swabs (Tables 2, 3).

### Neutralization test results

Anti-LSDV specific antibody titers were detected in the serum of virus-inoculated bulls No. 3, No. 5 and No. 7 from the IN-group, as well as the bulls from the contact IC-group (No. 4 and No. 6) starting from day 22 pi. The titers of the IN bulls were higher than for the IC group (Table 4). Bulls No. 1, No. 2, No. 8, No. 9 and No. 10 which represent both groups (IC and IN) were sero-negative during the whole experiment. Full information about results for all experimental bulls is illustrated in Table 4.

**Table 4:** Neutralization test results of sera from the experimental animals.

**No. bull**	**Antibody titers**						
	**Day post infection**						
	**1**	**6**	**15**	**22**	**29**	**36**	**41**
1	0	0	0	0	0	0	0
2	0	0	0	0	0	0	0
3	0	0	0	**1:128**	**1:256**	**1:256**	**1:256**
4	0	0	0	0	**1:8**	**1:16**	**1:16**
5	0	0	0	**1:64**	**1:64**	**1:128**	**1:128**
6	0	0	0	0	**1:64**	**1:32**	**1:32**
7	0	0	**1:16**	**1:128**	**1:64**	**1:128**	**1:128**
8	0	0	0	0	0	0	0
9	0	0	0	0	0	0	0
10	0	0	0	0	0	0	0

Bold means positive.

### Virus isolation

The causative agent LSDV was successfully isolated in cell culture from nasal swabs obtained from seven animals (Table 5). No viable LSDV was isolated from the nasal swabs of the infected animals No1 and No9 as well as the in-contact bull No10, over the 41 days of the experiment (Table 5). Similarly, viable virus was not isolated from biological materials of infected animals No1 and No9 as well as the the in-contact animals No8 and No10 (Table 6).

**Table 5:** LSD virus isolation in cell culture from nasal swabs of experimental animals.

**Day post infection (dpi)**	**Animal number**									
	**1**	**2**	**3**	**4**	**5**	**6**	**7**	**8**	**9**	**10**
1	Neg.	Neg.	Neg.	Neg.	Neg.	Neg.	Neg.	Neg.	Neg.	Neg.
3	Neg.	Neg.	Neg.	Neg.	Neg.	Neg.	Neg.	Neg.	Neg.	Neg.
6	Neg.	Neg.	Neg.	Neg.	Neg.	Neg.	Neg.	Neg.	Neg.	Neg.
7	Neg.	Neg.	Neg.	Neg.	Neg.	Neg.	Neg.	Neg.	Neg.	Neg.
10	Neg.	Neg.	Neg.	Neg.	Neg.	Neg.	Neg.	Neg.	Neg.	Neg.
13	Neg.	Neg.	**Pos**.	Neg.	**Pos**.	Neg.	**Pos**.	Neg.	Neg.	Neg.
15	Neg.	**Pos**.	**Pos**.	**Pos**.	**Pos**.	Neg.	**Pos**.	Neg.	Neg.	Neg.
17	Neg	**Pos**.	**Pos**.	**Pos**.	**Pos**.	Neg.	**Pos**.	Neg.	Neg.	Neg.
19	Neg	**Pos**.	**Pos**.	**Pos**.	**Pos**.	Neg.	**Pos**.	Neg.	Neg.	Neg.
20	Neg	**Pos**.	**Pos**.	**Pos**.	**Pos**.	Neg.	**Pos**.	Neg.	Neg.	Neg.
22	Neg	**Pos**.	**Pos**.	**Pos**.	**Pos**.	**Pos**.	**Pos**.	**Pos**.	Neg.	Neg.
24	Neg	**Pos**.	**Pos**.	**Pos**.	**Pos**.	**Pos**.	**Pos**.	**Pos**.	Neg.	Neg.
27	Neg	**Pos**.	**Pos**.	**Pos**.	**Pos**.	**Pos**.	**Pos**.	**Pos**.	Neg.	Neg.
29	Neg	**Pos**.	**Pos**.	**Pos**.	**Pos**.	**Pos**.	**Pos**.	**Pos**.	Neg.	Neg.
31	Neg	**Pos**.	**Pos**.	**Pos**.	**Pos**.	**Pos**.	**Pos**.	**Pos**.	Neg.	Neg.
34	Neg	**Pos**.	**Pos**.	**Pos**.	**Pos**.	**Pos**.	**Pos**.	**Pos**.	Neg.	Neg.
36	Neg	**Pos**.	**Pos**.	**Pos**.	**Pos**.	**Pos**.	**Pos**.	**Pos**.	Neg.	Neg.
38	Neg	**Pos**.	**Pos**.	**Pos**.	**Pos**.	**Pos**.	**Pos**.	**Pos**.	Neg.	Neg.
40	Neg	**Pos**.	**Pos**.	**Pos**.	**Pos**.	**Pos**.	**Pos**.	**Pos**.	Neg.	Neg.
41	Neg	**Pos**.	**Pos**.	**Pos**.	**Pos**.	**Pos**.	**Pos**.	**Pos**.	Neg.	Neg.

neg., viable LSD virus not isolated; pos., viable LSD virus isolated.

**Table 6:** LSD virus isolation in cell culture from pathological materials of experimental animals.

**Animal number**	**Type of pathological material**				
	**Lymph nodes**	**Skin**	**Nasal mucosa**	**Lungs**	**Skeletal muscle**
1	Neg.	Neg.	Neg.	Neg.	Neg.
2	**Pos**.	**Pos**.	**Pos**.	**Pos**.	Neg.
3	**Pos**.	**Pos**.	**Pos**.	**Pos**.	Neg.
4	**Pos**.	**Pos**.	**Pos**.	**Pos**.	Neg.
5	**Pos**.	**Pos**.	**Pos**.	**Pos**.	**Pos**.
6	**Pos**.	**Pos**.	**Pos**.	Neg.	Neg.
7	**Pos**.	**Pos**.	**Pos**.	**Pos**.	Neg.
8	Neg.	Neg.	Neg.	Neg.	Neg.
9	Neg.	Neg.	Neg.	Neg.	Neg.
10	Neg.	Neg.	Neg.	Neg.	Neg.

neg., viable LSD virus not isolated; pos., viable LSD virus isolated.

The results of virus isolation showed that viable LSD virus was isolated from different organs and pathological materials taken from animals in both groups, IN and IC, which indicated that the virus was transmitted from infected animals to animals in contact and successfully replicated. Viable virus could not be isolated from four animals (No.1, 8, 9, and 10), belonging to both groups, irrespective of the sample material used.

## Discussion

The conflicting data concerning the mode of LSDV transmission remains a significant gap in the current knowledge of the disease. Originally called pseudo-urticaria, it was suggested that the disease resulted from either poisonous plants or insect bites (15). The latter was the popular hypothesis, since outbreaks were confined to the moist, low-lying areas following copious rainfall and high humidity (24). Based on these historical observations, the spread of LSD has been associated with hematophagous insect activity, since LSD outbreaks predominantly occurred in the warm summer months following intensive rainfalls, when the insect numbers were at their highest (15). Thus, the transmission of LSDV has been suggested to occur *via* either direct or indirect contact and mechanically with the aid of insect vectors. Based on molecular PCR experiments, various insects have been associated with the presence of LSDV genomic material. These include flies, ticks and mosquitoes (25–28).

LSDV belongs to the genus *Capripoxvirus* that includes the two closely related SPPV and GTPV. The transmission of both SPPV and GTPV occurs predominantly through the inhalation of large airborne respiratory droplets of infectious virus, but alternative methods including contact *via* skin abrasions and nasal secretion, insects and fomites have been reported as well (29). The airborne mode of transmission is also the predominant method across the family *Poxviridae* (12). This challenges the hypotheses that LSDV is considered a vector-borne pathogen and additionally that contact transmission is ineffective. Haig reported in 1957 that even the use of insecticides had no effect on the spread of the disease that seems to follow the railway infrastructure of South Africa, alluding to some yet unknown factors driving the transmission (24).

The recent literature review on LSDV transmission highlights the focus of researchers on the vector-borne concept, but the global epidemiology of LSD is still poorly understood and cannot explain the occurrence of LSD outbreaks during the cold months under frozen conditions (21). Detailed studies into the molecular evolution of LSDV described the first observation of novel genomic lineages that include LSDV strains generated *via* homologous recombination between vaccine strains Neethling (major parent) and Kenyan (minor parent). These circulating novel recombinant strains emerged in the face of a vaccination campaign using contaminated vaccines (30). Seen as a mechanistic by-product of the recombination machinery to repair broken replication forks during genome replication, the resulting novel recombinant strains turned out to possess specific genomic regions under selection (18). The naturally occurring recombinant vaccine-like strains are currently the dominant lineage in Asia, demonstrating unique properties allowing them to overwinter and spread during freezing winters (18).

Udmurtiya/2019 is a unique strain in that: i) it caused an outbreak during a snowy month in Russian temperate climate, which had never been observed; ii) the parental strain contributions are reversed compared to Saratov/2017, i.e., the major backbone is the Kenyan vaccine strain with insertions of the Neethling-LW1959 vaccine strain (21). These novel circumstances warranted a study to investigate its pathogenesis and transmission and identify how it correlates to the vaccine-like recombinant Saratov/2017, whose transmission without insect vectors has been already proven (16).

In this study, we investigated the ability of another recombinant vaccine-like strain Udmurtiya/2019 to be transmitted through an indirect contact route in cattle. The animals were housed in the same ventilated room, but were restricted to have no physical face-to-face contact and were provided with separate feeding, bedding and watering stations. Half of the animals were infected and shared the same airspace with non-infected animals and since representatives of both groups of animals developed LSD, it clearly indicates an alternative indirect contact mode of transmission. In-contact transmission between infected and uninfected animals was suggested when a classical LSDV field strain (LSDV-V/281-Nigeria) was used as an inoculum (31). The authors reported PCR positive results from nasal swabs from the in-contact animals, but these animals did not produce any clinical signs of viral infection, nor had viraemia detected from blood, nor seroconverted, nor were virus isolation attempted (31). Since there is no scientific justification to suggest that poxviruses display local replication without disseminating *via* the lymph and blood (32), additional studies are required to clarify this issue concerning classical LSDV strains that have thus far been principally associated with transmission *via* insects. It is important to further examine the reasons contributing to the previously published in-contact animals not resulting in subsequent infection, despite indicating exposure to the virus i.e., required infective viral load, genetic composition of the virus, health, age and breed of the animal or time of the trial as previously suggested (31). Currently, contact transmission has been conclusively established for recombinant vaccine-derived LSDV strains that demonstrate the presence of viral DNA in both nasal secretions as well as at the viraemic stage (Tables 2, 3) (16).

The genomic composition of each of the five novel recombinant LSDV strains have been described and compared in detail recently (18). Additionally, the open reading frames (ORF) and single nucleotide polymorphisms (SNPs) positively selected for in these five recombinant strains have been identified and described (18), but in order to link these genotypes to functional activities, possible differences in the phenotype of these recombinant strains should be identified. It is therefore imperative that the different phenotypic characteristic between the novel recombinant strains as well as the differences between the recombinants and the classical strains be identified in order to link future targeted mutational genotypes to these phenotypes. In a previous experiment, virulent Saratov/2017 with the genomic backbone of a Neethling commercial vaccine, first demonstrated the potential for non-vector borne transmission and in a subsequent study it was shown to cause a mild infection *via* the alimentary route (19). By contrast, Udmurtiya/2019 with the genomic backbone of a Kenyan vaccine strain KSGP showed a similar characteristic. Considering that the experimental animals shared only the same ventilated air without sharing troughs, both Saratov/2017 and Udmurtiya/2019 could be transmitted through an indirect contact route (19). This is not surprising because closely related SPPV and GTPV are readily transmitted through close contact and in contaminated environments *via* fomites contaminated by oronasal secretions produced by acutely infected animals (29). Aerosols are an important route of poxvirus transmissions, with examples of myxoma virus, which is mechanically transmitted by mosquitoes, spreading *via* direct contact (33). Since Udmuritya/2019 was recovered during a snowy and freezing winter in northern latitudes, contact transmission is conclusively established in this case both in the field and experimentally (21).

Interestingly, despite being inoculated with the virus, bull No. 1, remained negative for the majority of the study period whilst testing transiently positive on day 20 pi (Tables 2, 3). This is important because this intravenously infected bull did not succumb to infection, having demonstrated subclinical infection. It appears if the animal promptly cleared the virus and looked healthy until the end of the experiment. This observation could be associated with the genetics, age and physical condition of the host as well as the dose or route of inoculation conferring resistance. Subclinical infection of experimentally infected animals have been described for LSD, regardless of the virus lineage (19, 31, 34, 35). In this regards, animals infected with a recombinant vaccine-like without overt symptoms do pose a risk of transmission in contrast to the vector-borne classical isolates (19, 31, 34). This merits further investigation.

Similar clinical symptoms were observed when studying the two novel recombinant strains of LSDV under experimental conditions. In this work, Udmurtiya/2019 exhibited prolonged virus shedding in blood and nasal secretions for more than 30 days, whilst Saratov/2017 was detected in blood for the same period, but nasal shedding lasted for only 27 days (36). In both these cases the observed shedding patterns of recombinant strains were three times longer than for classical LSDV isolates, both in blood and nasal swabs (29, 36). Since these experiments were carried out independently with slightly different study designs, the direct comparison might not be accurate. In this regard more comparative studies using recombinant and classical field strains in standardized experimental conditions should be conducted to determine if these are unique features of the novel recombinants. However, such a study involving different lineages of LSDV Dagestan/2015 and Saratov2017 under the identical conditions has been published, but another for Udmurtiya is required (19). It was indicated that recombinant Saratov/2017 does exhibit unique patterns of alternative transmission versus the classical Dagestan2017 strain (19).

In addition to these observations, Saratov/2017 demonstrated a more aggressive CPE pattern in an *in vitro* study (37), thus providing evidence that genomic alterations did provide significant novel pathogenic features to the resulting viruses.

An increasing number of studies are devoted to examining the genomic composition of circulating and archived LSDV strains, with low genetic diversity described within their sequence identity (38). Prior to 2017, experiments were based on classical field strains whose contact transmission was arguable, ineffective and provided strong support to the vector-borne concept that is enjoying rising attention (39). Since the first identification of novel recombinant vaccine-like strains and their establishment as the dominant lineage in South East Asia, the current approaches to risk assessment and control strategies need revisiting (18).

Overall, our results clearly demonstrate non-vector borne transmission for the recombinant strain Udmurtiya/2019 and lend support that LSDV can be transmitted by indirect contact routes similar to related *capripoxviruses*. Considering that recombinant vaccine-like strains of LSDV are on the rise in South Eastern Asia, additional studies into alternative transmission patterns are warranted to gain as much knowledge as possible on the global epidemiology of LSD.

## Data availability statement

The original contributions presented in the study are included in the article/supplementary material, further inquiries can be directed to the corresponding author.

## Ethics statement

The animal study was reviewed and approved by Committee of the Federal Center for Animal Health, Russia (Permit Number: No. 2/1-21082018) and conducted in strict accordance with Directive 2010/63/EU on the protection of animals used for scientific purposes.

## Author contributions

AN, IS, AK, VK, ID, and AS designed the experiments and collected the data. AM, OB, AV, IC, and AS analyzed the data and wrote the manuscript. All authors read and approved the final version of the manuscript.

## Funding

This work was supported by the Grant No. 075-15-2021-1054 from the Ministry of Education and Science of Russia to implement objectives of the Federal Scientific and Technical Program for the Development of Genetic Technologies during 2019–2027.

## Conflict of interest

The authors declare that the research was conducted in the absence of any commercial or financial relationships that could be construed as a potential conflict of interest.

## Publisher's note

All claims expressed in this article are solely those of the authors and do not necessarily represent those of their affiliated organizations, or those of the publisher, the editors and the reviewers. Any product that may be evaluated in this article, or claim that may be made by its manufacturer, is not guaranteed or endorsed by the publisher.
